# Supplementary material for: Coupling Plasmonic and Cocatalyst Nanoparticles on N–TiO2 for Visible-Light-Driven Catalytic Organic Synthesis
Source: Nanomaterials (Basel). 2019 Mar 7;9(3):391. doi: 10.3390/nano9030391 (PMC6473962; doi:10.3390/nano9030391)
Supplement: Supplementary file 1 [file nanomaterials-09-00391-s001.pdf]

Coupling Plasmonic and Cocatalyst Nanoparticles on N–TiO_2 for Visible-Light-Driven Catalytic Organic Synthesis

Yannan Wang, Yu Chen, Qidong Hou, Meiting Ju and Weizun Li *

College of Environmental Science and Engineering, Nankai University, Tianjin 300350, China;

Experimental

1. Synthesis

Synthesis of TiO_2 nanocrystals with a hollow structure. TiO_2 nanocrystals were prepared according to our previous paper [1]. In a typical procedure, 3.54 mL of acetic acid, 0.35 mL of water, and 4.372 mL of 1-butyl-3-methylimidazolium tetrafluoroborate ([Bmim][BF_4], Sigma-Aldrich, Shanghai, China) were first mixed in a Teflon beaker. Secondly, tetrabutyl titanate was added to the solution under magnetic stirring, affording a transparent solution. Finally, the homogeneous solution was transferred to a 70-mL Teflon-lined autoclave, which was then placed in a microwave–solvothermal synthesis system (MDS-6, Sineo, Shanghai, China) and maintained at 200 °C for four hours. After cooling to room temperature, the resulting powder was separated by centrifugation, washed several times with ethanol and deionized water, and dried at 60 °C for 12 h.

Nitrogen doping. The pristine TiO_2 product was mixed and ground with urea (1:4), followed by heating the mixture at 400 °C for two hours in air to obtain N–TiO_2 [2].

Photodeposition of noble metal. Noble metals were deposited on N–TiO_2 nanocrystals by a typical photo-deposition–calcination method. The deposition of Au and Pt were achieved using chloroauric acid ($HAuCl_4$, Sigma-Aldrich) and hexachloroplatinic (IV) acid (H_2PtCl_6, Macklin, Shanghai, China) as the precursors. Typically, for the Au_2Pt_2/N-TiO_2 (denoted as 2 wt.% Au and Pt) sample, 4.125 mL of $HAuCl_4$ (10 g/L), and N–TiO_2 (1 g) were first dispersed in a 40-mL methanol solution in a Pyrex glass reactor. Secondly, the mixture was subjected to UV-light irradiation for two hours using a high-pressure mercury lamp (300 W) as the light source. Before irradiation, gaseous Ar was bubbled through the mixture for 30 min to verify the absence of dissolved O_2 in the solution. During photo-deposition, the color of the suspension gradually changed from white to auburn, indicative of the reduction of the Au precursors. Pt NPs serving as the cocatalyst were deposited by in situ photo-deposition. After loaded gold nanoparticles, the cocatalyst source (Pt precursor 5.187 mL of H_2PtCl_6 (10 g/L)) was injected into the sealed tube, and then gaseous Ar was bubbled through the mixture for 30 min. The Pt precursor of the cocatalyst was reduced by photogenerated electron reduction using a 300-W high-pressure mercury lamp, and magnetically stirred for 2 h in a water bath at a temperature of 25 °C. Then, the powder was collected by centrifugation and washed several times with ethanol and water and dried at 80 °C. Finally, the products were annealed in air at 350 °C for five hours for further characterization and use.

2. Sample Characterization.

X-ray diffraction (XRD) patterns were recorded on a PANalytical X'pert MRD system with a step size of 0.02° at 2θ values of 10°–80° to identify the TiO_2 and noble-metal phases. Diffuse-reflectance UV–Vis spectra of the samples (ca. 20 mg of sample diluted in ca. 80 mg of $BaSO_4$) were recorded on a Shimadzu 3600 UV–Vis spectrophotometer in air against $BaSO_4$ from 300 to 800 nm. Scanning electron microscopy (SEM) images were recorded on a Hitachi S4800 microscope to observe the morphologies of the TiO_2 composites. Transmission electron microscopy (TEM) images were recorded on an FEI Tecnai F20 microscope at an accelerating voltage of 200 kV. X-ray photoelectron spectra (XPS) of the samples were recorded on a Thermo Fisher Scientific ESCALAB 250XI system using a monochromatic Al-Ka X-ray source. Accurate binding energies (± 0.1 eV) were determined

concerning the position of the adventitious C *1s* peak at 284.6 eV. Room-temperature photoluminescence (PL) spectra were recorded on a fluorescence spectrophotometer (F-7000, Tokyo, Japan) at an excitation wavelength of 250 nm and a scanning speed of 1200 nm/min. Specific surface areas were estimated by N_2 adsorption/desorption isotherms recorded at −196°C on a TriStar II 3020 adsorption analyzer. The samples were degassed under vacuum at 200 °C for four hours before analysis. Photocurrent and electrochemical impedance spectroscopy (EIS) measurements were carried out in a standard three-electrode cell containing a 0.5 mol·L^{-1} Na_2SO_4 aqueous solution with a platinum foil and saturated calomel electrode as the counter and reference electrodes, respectively, on a CHI 760D workstation (Shanghai, China). A 300-W Xe lamp with a 460-nm cut-off filter was selected as a visible-light source. All electrochemical measurements were carried out at room temperature.

3. Photocatalytic Activity Measurement

The photocatalytic selective oxidation of alcohols was carried out in a 15-mL homemade reactor sealed with rubber stoppers. Typically, 10 mg of photocatalyst was spread into the reactor, followed by the injection of 1.5 mL of trifluorotoluene (solvent) and 0.1 mmol of benzyl alcohol (reactant) into the reactor. Secondly, the bottle was filled with molecular oxygen for 5 min and sealed with a rubber septum cap. Thirdly, the container was subjected to ultrasonication at room temperature for 5 min to distribute the catalyst in the solution evenly. The reactor was subjected to photoirradiation under magnetic stirring using a 300-W xenon lamp (PLS-SXE 300, Beijing Perfectlight, Co., Ltd., Beijing, China) with a UV-cut filter to provide light with wavelengths $\lambda > 460$ nm. The photocatalytic reaction temperature was controlled at room temperature using a water-cooling system. Control photoactivity experiments using different radical scavengers (such as ammonium oxalate (AO) as the scavenger for photogenerated holes; *tert*-butyl alcohol (TBA) as the scavenger for hydroxyl radicals; $AgNO_3$ as the scavenger for electrons; and benzoquinone (BQ) as the scavenger for superoxide radical species) were carried out in the same manner as those for the abovementioned photocatalytic oxidation of benzyl alcohol except that radical scavengers (0.1 mmol) were added into the reaction system. After the reactions, the organic products were analyzed by gas chromatography (Thermo Fisher, Waltham, MA, USA, TRACE 1310GC) equipped with a flame ionization detector. GC–MS analysis on a Thermo Fisher TRACE ISQ GCMS system, which was equipped with a TR–50MS capillary column (Thermo Fisher, 30 m × 0.320 mm × 0.25 µm), was employed for product identification. The column oven temperature was programmed from 40 °C (held for 1 min) to 280 °C at a rate of 40 °C·min^{-1}. After the reaction, the catalyst was recovered by careful washing with anhydrous ethanol and deionized water and dried overnight in an oven at 80 °C for the following cycling photoactivity test. The alcohol conversion and aldehyde yield were defined as follows:

$$Conversion\ (\%) = \left[\frac{C_0 - C_{alcohol}}{C_0}\right] \times 100$$

$$Yield\ (\%) = \frac{C_{aldehyde}}{C_0} \times 100$$

$$Selectivity\ (\%) = [C_{aldehyde}/(C_0 - C_{alcohol})] \times 100$$

where C_0 represents the total amount of alcohols in the solution before illumination, $C_{alcohol}$ represents the amount of alcohols in the solution after illumination for 2.5 h, and $C_{aldehyde}$ represents the amount of aldehyde in the solution, and after illumination for 2.5 h.

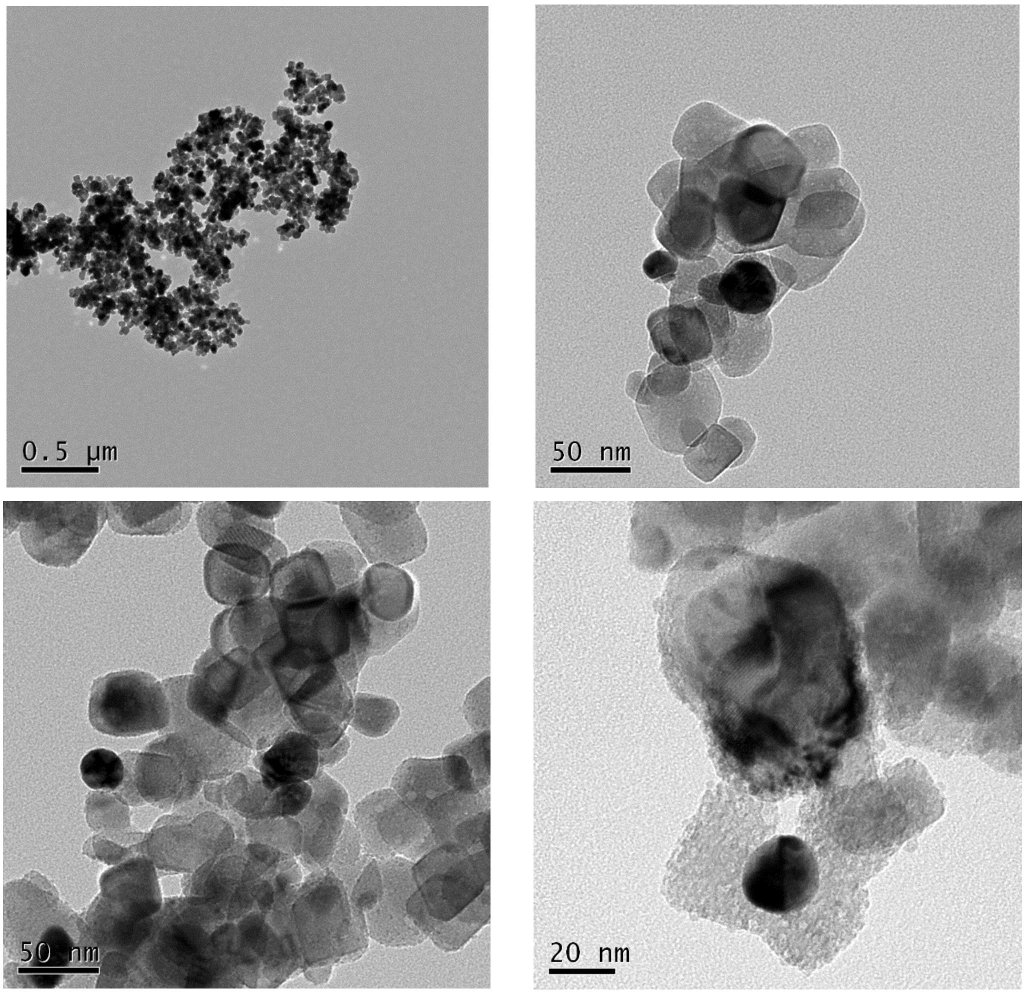

Figure S1. TEM images of $Au_2Pt_2/N–TiO_2$ photocatalyst.

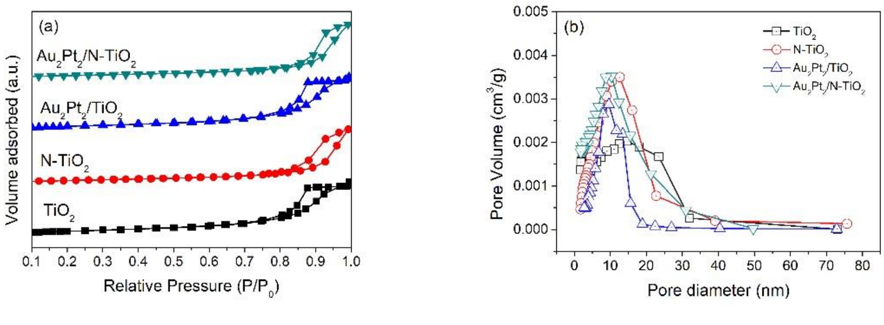

Figure S2. (**a**) N_2 adsorption/desorption isotherm of as-prepared catalysts; (**b**) the corresponding Barrett–Joyner–Halenda (BJH) desorption pore size distribution.

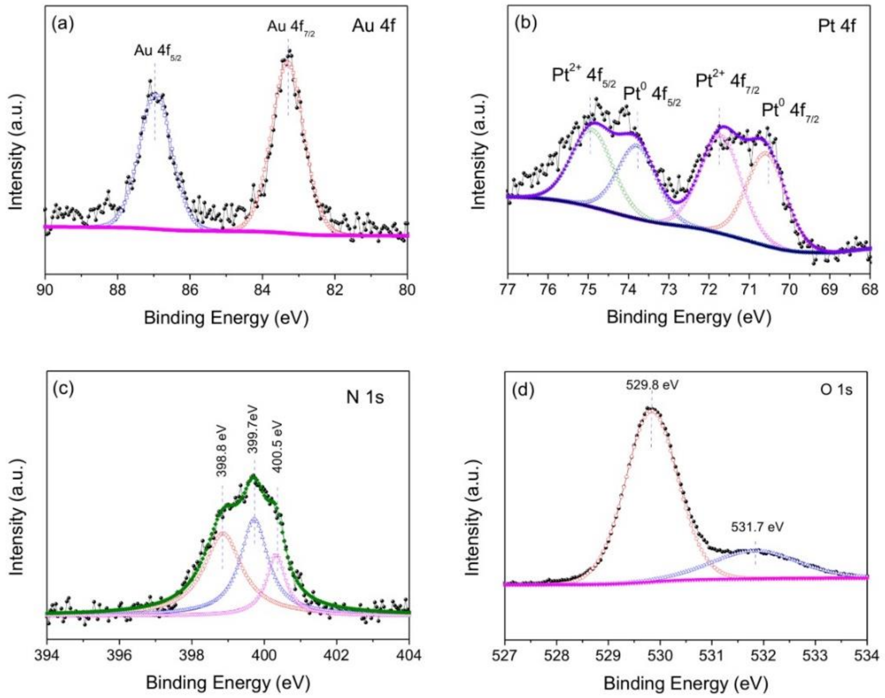

Figure S3. Fine XP spectra of (**a**) Au *4f*, (**b**) Pt *4f*, (**c**) N *1s*, and (**d**) O *1s* obtained from $Au_2Pt_2/N\text{-}TiO_2$.

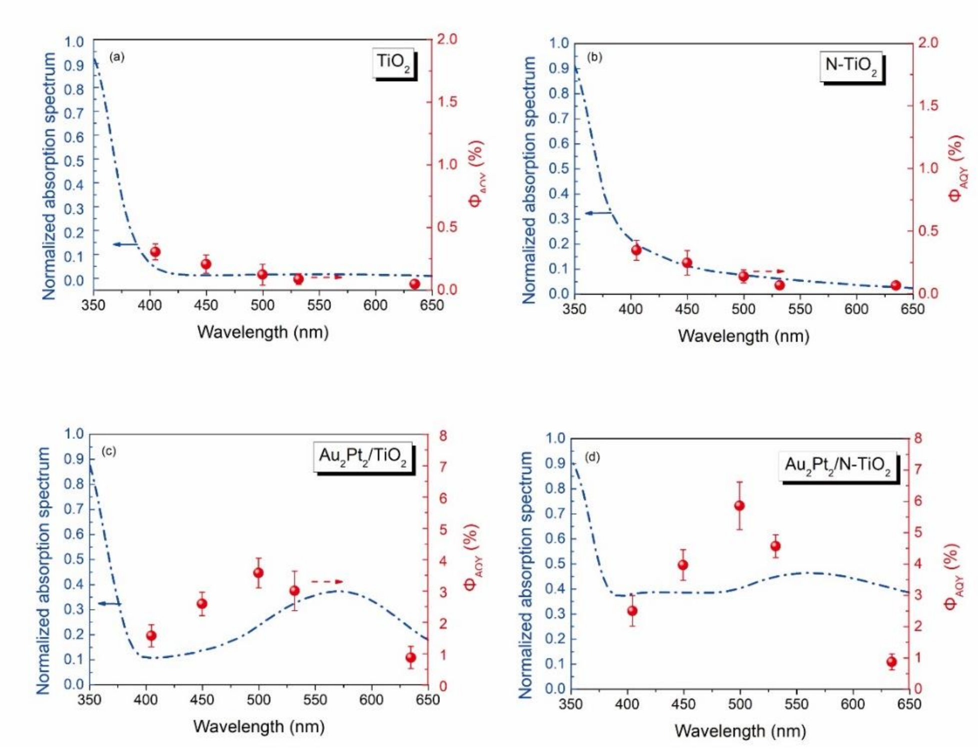

Figure S4. (**a–d**) Diffuse-reflectance UV–Vis spectra of TiO_2, $N\text{-}TiO_2$, Au_2Pt_2/TiO_2, and $Au_2Pt_2/N\text{-}TiO_2$ catalysts and the quantum yield for the formation of benzaldehyde under laser irradiation of different wavelengths, such as 405 nm, 450 nm, 500 nm, 532 nm, and 635 nm. The apparent quantum yield was calculated using the equation $\Phi_{AQY} = (Y_{vis} - Y_{dark})/N \times 100\%$, where Y_{vis} and Y_{dark} denote the yield of benzaldehyde under light and dark conditions, respectively. N denotes the number of incident photons in the reaction vessel.

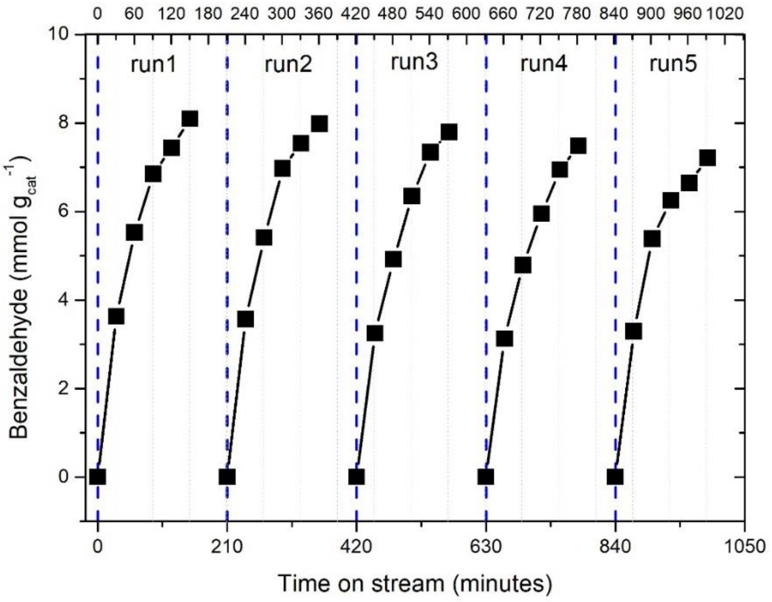

Figure S5. Recyclability tests of $Au_2Pt_2/N–TiO_2$ for the selective oxidation of benzyl alcohol.

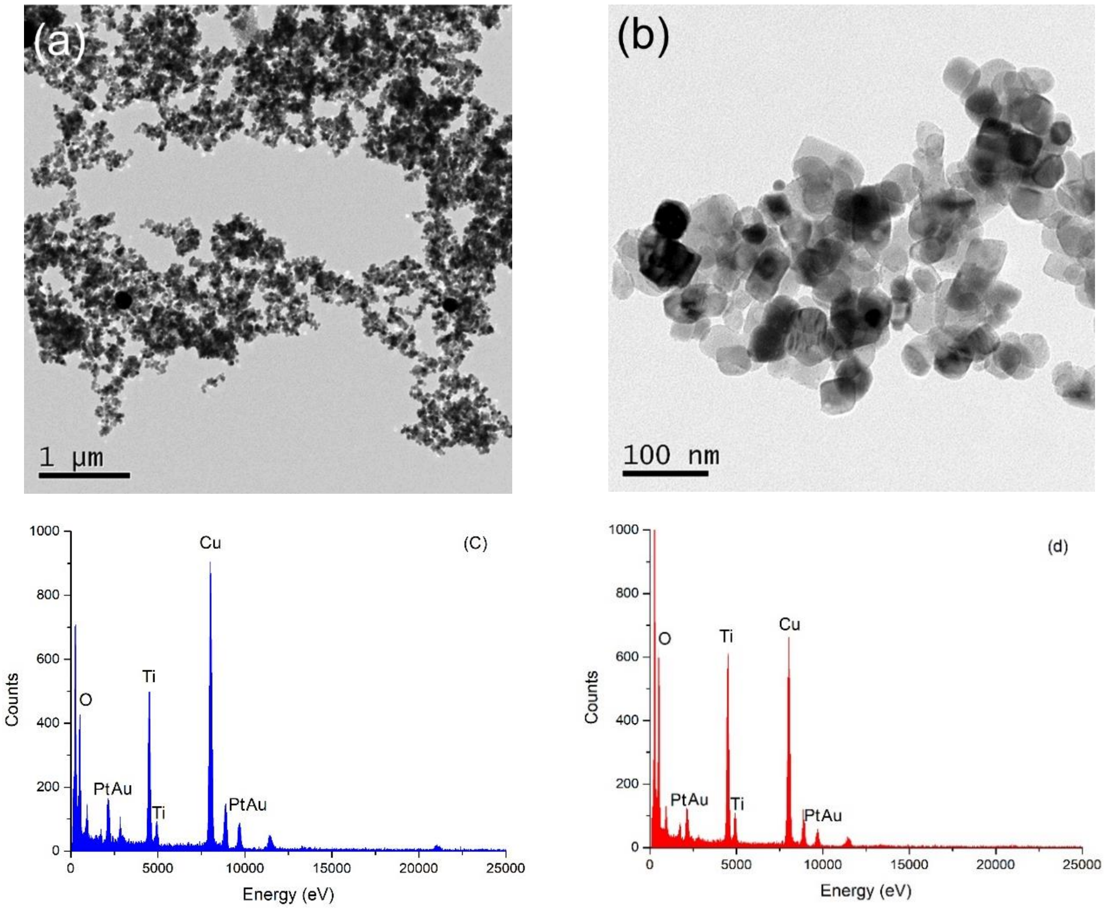

Figure S6. (**a**,**b**) TEM images of $Au_2Pt_2/N–TiO_2$ catalyst after photocatalysis reaction; (**c**,**d**) the EDX analysis of photocatalysis before and after photocatalysis reaction.

Table S1. Summarized physical and chemical data for TiO_2, N–TiO_2, Au_2Pt_2/TiO_2, and Au_2Pt_2/N–TiO_2 photocatalysts.

Sample	Metal content [a] (wt.%)	S_{BET} [b] (m^2/g)	Pore size [c] (nm)	Pore volume [d] (cm^3/g)
TiO_2	0.00	57.77	12.14	0.36
N–TiO_2	0.00	42.84	10.97	0.31
Au_2/N–TiO_2	1.87	39.15	10.13	0.28
Pt_2/N–TiO_2	1.76	41.51	10.46	0.29
Au_2Pt_2/N–TiO_2	1.86 (Au); 1.72 (Pt)	39.04	10.01	0.29
Au_2Pt_2/TiO_2	1.83 (Au); 1.67 (Pt)	54.83	11.15	0.31

[a] Weight percentage of Au and Pt measured by EDS.

[b] S_{BET}, Brunauer–Emmett–Teller (BET) surface areas calculated by the adsorption branch of the N_2 isotherm.

[c] D_{BJH}, pore diameter determined by Barrerr–Joyner–Halenda (BJH) model.

[d] V_t, total pore volume.

References

1. Chen, Y.; Li, W.; Wang, J.; Gan, Y.; Liu, L.; Ju, M. Microwave-assisted ionic liquid synthesis of Ti^{3+} self-doped TiO_2 hollow nanocrystals with enhanced visible-light photoactivity. *Appl. Catal. B Environ*. **2016**, *191*, 94–105.
2. Wang, W.; Lu, C.; Ni, Y.; Su, M.; Xu, Z. A new sight on hydrogenation of F and N-F doped {001} facets dominated anatase TiO_2 for efficient visible light photocatalyst. *Appl. Catal. B Environ*. **2012**, *127*, 28–35.
